# Clinical pictures mimicking acute hernia complications: are we really daring to violate a traditional abdominal wall surgery dogma?

**DOI:** 10.1186/s12893-020-00899-1

**Published:** 2020-10-15

**Authors:** Michela Zanatta, Giovanna Brancato, Guido Basile, Francesco Basile, Marcello Donati

**Affiliations:** 1 Surgical Clinic Unit, Department of Surgery and Medical-Surgical Specialties, University of Catania, 95123 Catania, Italy; 2 Emergency and Abdominal Surgery Unit, Department of Surgery and Medical-Surgical Specialties, University of Catania, Via Santa Sofia 78 CT, 95123 Catania, Italy

**Keywords:** Acute hernia complications, Hernia fistulization, Hernia strangulation, Conservative treatment, Case report

## Abstract

**Background:**

Acute abdominal wall hernia complications usually require a prompt surgical treatment. The aim of this case series is to report our experience with some unusual cases of apparent acute and subacute hernia complications not requiring surgical treatment, changing the classical paradigm of immediate surgical approach into a “wait and see” situation.

**Case presentation:**

We shortly report here four cases of abdominal wall hernia complications in which surgical treatment could have been unsafe for the patients considering their clinical condition. Two cases were fistulated and two were apparently strangulated. After clinical evaluation and CT-scan, we opted for a conservative treatment weighting the risk–benefit balance in order to give the best quality of life to the patient.

**Conclusions:**

In selected cases and under well-defined situations, an accurate evaluation should convince every surgeon to opt for a conservative approach refraining from a promptly operative treatment of the patient. This may be particularly relevant among very old or high-risk patients affected by long-standing abdominal wall hernias.

## Background

Acute or subacute abdominal wall hernia complications usually require surgical treatment [1]. The aim of this work is to report our experience with acute and subacute hernia complications that did not require surgical treatment.

## Case presentation

*Case 1* An 82 year old female came to our observation during a familiar house consulting (the patient was in an elderly patient private institution for chronic diseases). Surgical consultation was induced by a sudden spontaneous emission of faeces from an inveterate long-standing, non-reducible umbilical hernia for at least 20 years. The umbilical hernia was never painful and no anamnestic episodes of strangulation or bowel paralysis together with nausea and vomit were recorded. The patient was completely apyretic, with normal bowel function (normal gas emission and peristaltism); no acute abdomen symptoms or any other symptomatology were referred other than spontaneous bowel content exit from the umbilical area over the palpable non-reducible hernia. By physical examination the skin appeared to be enflamed due to the local irritating action of faeces around the cutaneous orifice. We opted for conservative treatment. The patient died of cardiorespiratory complications due to prolonged bed immobilization, 2 years and 6 months after our observation. Oral intake and any feeding were regular until the end, as well as the physiologic canalization. The “spontaneous colostomy” worked correctly until the end. No symptoms or problems were reported from the umbilical hernia.

**Fig. 1:**
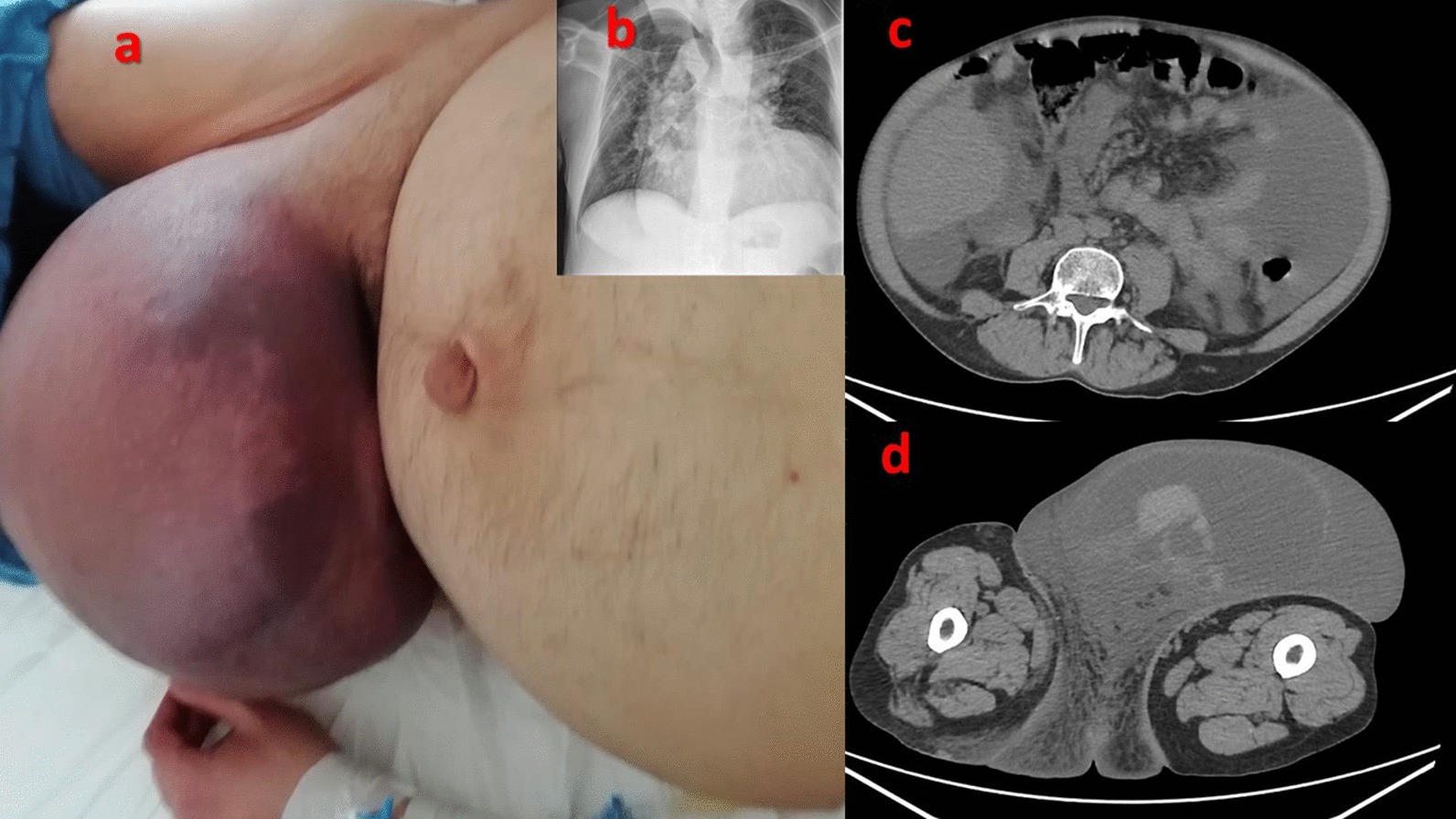
Case 2. **a** Giant inguino-scrotal hernia apparently strangulated. **b** Thoracic X-ray showing cardiomegaly (right ventricle hypertrophy). **c** CT-scan showing copious ascitic fluid in abdomen. **d** CT-scan ruling out intestinal strangulation

*Case 2* A 40 year old male patient with a well-known giant inguino-scrotal hernia came to our observation during an episode of severe abdominal pain and paralytic ileum. The clinical exam pointed out tumefaction and inflammation of scrotal region, compatible with the diagnosis of strangulated hernia. Anamnestically the patient underwent surgery in 1991 and 1995 for a congenital cardiopathy probably related to DiGeorge Syndrome; he also had heart failure (NYHA score III), a permanent atrial fibrillation, pulmonary valve insufficiency, thrombocytopenia and phlebostatic ulcers due to lower limbs chronic insufficiency. He assumed anticoagulant therapy. During physical examination the hernia sack appeared to be non-reducible without evocated pain (Fig. 1a). He underwent CT scan without contrast agent (due to chronic kidney failure) which highlighted the presence of a hematoma in inguino-scrotal region and hepatomegaly with copious ascitic fluid (Fig. 1b). We opted for a conservative treatment: during hospitalization he started fasting and underwent a cardiologic consultancy to adjust the anticoagulant therapy. The patient was discharged after modifying his cardiologic therapy. To date, the patient’s clinical course was uneventful and he was not operated for his inguino-scrotal hernia.

*Case 3* [2] A 78 year old male patient came to our observation referring a long-standing bilateral inguinal hernia, extending down to the knee on the right side. The hernia sack was irreducible although the patient did not complain about altered bowel function; he referred a weight loss of about 8 kg in 6 months. In consideration of the particular clinical situation, we opted for a CT-scan evaluation that highlighted the presence of a giant multilobular tumor with the major diameter of the biggest nodule of 27 cm. To better identify the tumor we performed a CT-guided biopsy that revealed the presence of a GIST. We opted for Imatinib therapy with great results: the tumor reduced its volume after three months of therapy and became technically resectable. The patient refused surgery due to the high risk of complications. After a 5-year follow-up the patient is in good health and still under Imatinib therapy.

**Fig. 2:**
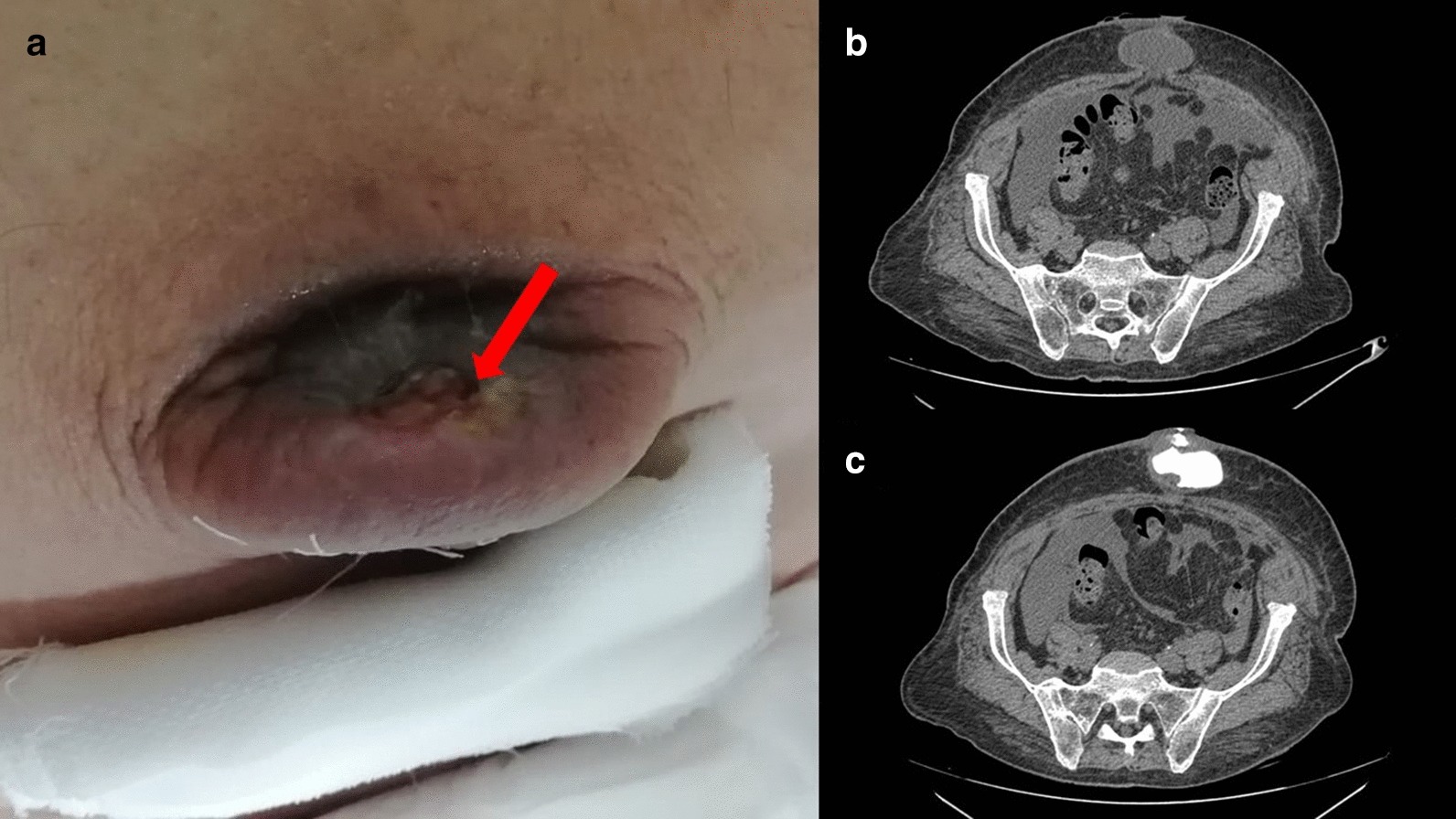
Case 4. **a** Fistulization of umbilical hernia. Red arrow indicates the cutaneous orifice. **b** CT-scan showing the umbilical hernia sack with a fluid content. **c** CT-scan with fistulography showing the diffusion of the contrast agent in the peritoneal cavity from the cutaneous orifice., ruling out intestinal loop fistulization

*Case 4* A 69 year old male patient came to our observation complaining about the output of yellow corpuscular fluid material from the umbilical region. The patient had a permagna inguinal hernia and a small umbilical hernia which seemed to be fistulated (Fig. 2a). He had been previously hospitalized in the Department of Internal Medicine for non-alcoholic liver cirrhosis and ascites. In our Department the patient underwent a CT scan with the injection of Gastrografin to study the fistulous canal and establish the correct treatment (Fig. 2b). The CT scan did not point out a fistulization of the hernia’s content but just a fistulization with the peritoneal cavity (hernia sack). To improve the patient’s quality of life, we opted for positioning a colostomy bag in the site of the fistulated skin, whose fluid output was not daily but every other day (about 40 cc). He was discharged after a few days and admitted to the Department of Internal Medicine for the treatment of his hepatopathy. To date, the patient is in good clinical conditions and was not operated for his umbilical hernia.

## Discussion and conclusions

Acute hernia complications include incarceration, obstruction, strangulation and, although more rarely, fistulization and evisceration [3]; those conditions need a prompt treatment in order to prevent or reduce intestinal sufferance [4]. In our reported series we did not operate those four patients despite the apparent presence of an acute complication. In the first case the patient had a long-standing umbilical hernia which became non-reducible and fistulated with the skin: in consideration of the absolute absence of other symptoms, such as paralytic ileum or any abdominal pain or general septic condition, and of the high comorbidity (Chronic ischemic cardiac disease and Alzheimer’s disease), we were convinced to opt for conservative treatment. In the second case the patient had a non-reducible hernia, with paralytic ileum and fluid in abdomen: apparently a strangulated hernia; however, the results of the CT scan did not demonstrate intestinal sufferance. By a more thorough examination, we noticed that the redness of the skin above the hernia sack (Fig. 1a) was not due to a hernia complication but to a hematoma caused by anticoagulant therapy. The abdominal pain was due to the heart failure which had caused abundant abdominal effusion, also responsible of the paralytic ileum and of the pseudo-acute abdomen. Therefore, in consideration of the non-critical surgical situation of the patient and the high surgical risk due to comorbidities, we opted for a conservative treatment. In the third case the content of hernia sack was a giant GIST tumor. Because of the recovered normal bowel function and of the great results of chemiotherapy in reducing the tumor volume, we successfully opted for medical treatment [2]. In the fourth case the patient suffered for portal hypertension and the fistulization of the peritoneum was a natural way to drain the ascitic fluid outside and to decrease pressure, acting as a natural intermittent paracentesis. For this reason, we considered not to operate the patient, also taking into consideration his clinical condition.

Looking at the above-reported clinical situations, the surgeon could be instinctively induced to immediately operate those patients, focusing his attention at first on the apparent acute local complication. However, considering the general situation and maintaining a holistic vision of the patient, an accurate evaluation should convince every surgeon to opt for a “wait and see” approach and not to promptly operate the patient. This may be particularly relevant among very old or high-risk patients affected by long-standing abdominal wall hernias. The clinical course of those patients ruled in favour of our not easy clinical balance; therefore, we decided to share our experience with the surgical community.

Furthermore, cases number 2 and 4 could, maybe in the future, after bettering of general conditions, be suitable for a definitive surgical treatment in elective care.

An immediate surgical approach would be deleterious for all of the patients. Luckily none of the patients presented with a real strangulation otherwise a non-operative treatment would have been quite dangerous.

This report is clearly showing that, under well-defined clinical circumstances and in high-risk patients, the surgeon should refrain from surgically treating the above-mentioned clinical situations mimicking acute hernia complications, therefore only apparently violating a traditional dogma of hernia surgery.

## Data Availability

Data sharing is not applicable to this article as no datasets were generated or analysed during the current study.

## Abbreviations

NYHA: New York Heart Association
CT-scan: Computed Tomography scan
GIST: Gastrointestinal Stromal Tumor
